# Phosphate-solubilizing *Bacillus subtilis* Y31 promotes cucumber growth and yield: insights from rhizosphere microbiomics and bacterial genomics

**DOI:** 10.3389/fmicb.2025.1751005

**Published:** 2026-01-13

**Authors:** Yu Fu, Kaixin Lin, Bo Cheng, Lianfen Qi, Qingyin Zhang, Haokai Li, Xiaobo Chen, Chunxiao Zhang

**Affiliations:** 1 College of Food Science and Biology, Hebei University of Science and Technology, Shijiazhuang, China; 2 Fermentation Technology Innovation Center of Hebei Province, Hebei University of Science and Technology, Shijiazhuang, China; 3 Shijiazhuang Academy of Agriculture and Forestry Sciences, Shijiazhuang, China

**Keywords:** *Bacillus subtilis*, cucumber, genomic analysis, phosphate-solubilizing bacteria, rhizosphere microbiomics

## Abstract

Phosphate-solubilizing bacteria (PSB) play a vital role in sustainable agriculture by enhancing plant growth and improving crop yield. In intensive cucumber (*Cucumis sativus* L.) cultivation systems, soil degradation commonly occurs, making the optimization of phosphorus availability a key strategy for increasing production. However, studies examining the growth-promoting effects of PSB in cucumber remain limited. In this study, we isolated a novel PSB strain, Y31, from cucumber rhizosphere soil and identified it as *Bacillus subtilis*. Strain Y31 demonstrated the ability to solubilize calcium phytate and calcium phosphate, secrete multiple enzymes, produce siderophores, and exhibit antagonistic activity against pathogenic fungi. Inoculation with Y31 significantly promoted cucumber growth, increasing greenhouse yield by 35.30%. Notably, Y31 application increased soil available phosphorus and altered the abundance of soil fungal community. It reduced the relative abundance of *Botryotrichum* and *Chrysosporium*, while promoting the relative abundance of the *Penicillium* genus. Genome sequencing of *B. subtilis* Y31 revealed the presence of genes involved in phosphorus cycling, carbohydrate-active enzymes, and the biosynthesis of 10 secondary metabolites. Together, these findings indicated that *B. subtilis* Y31 enhanced cucumber growth and yield by improving phosphorus availability, modulating microbial community structure, and carrying gene clusters linked to phosphorus solubilization and plant growth promotion. Therefore, this study provided a basis for the efficient utilization of phosphorus resources and supported the development of sustainable agricultural practices.

## 1 Introduction

Phosphorus is an essential macronutrient required for plant growth and development (Lambers, 2022; Yang S. Y. et al., 2024; Yang F. et al., 2024; Zhao et al., 2024). Although total phosphorus in soils is generally abundant, most of it occurs in insoluble forms that are unavailable for direct plant uptake and utilization (Shen et al., 2011). Insufficient phosphorus availability severely restricts crop growth, leading to stunted development, premature abscission of reproductive organs, and substantial yield losses in agricultural systems (Dissanayaka et al., 2021; Lu et al., 2023; Zeng et al., 2025). To satisfy crop phosphorus demands, the application of phosphorus fertilizers is widely practiced. However, these inputs are rapidly immobilized through soil fixation or chelation with divalent cations, forming insoluble compounds that limit nutrient bioavailability (Elser, 2012). This not only reduces fertilizer use efficiency but also contributes to environmental issues such as water eutrophication and significant resource waste (Conley et al., 2009). Therefore, developing environmentally sustainable and economically viable phosphorus activation strategies is critical for advancing green agriculture (Trivedi et al., 2020; Copeland et al., 2025).

Phosphorus-solubilizing bacteria (PSB), a representative group of plant growth-promoting rhizobacteria, offer an effective biological approach for enhancing phosphorus bioavailability (Alori et al., 2017; Elhaissoufi et al., 2021). PSB secrete various enzymes, including phytases and phosphatases, which facilitate the degradation of organic phosphorus compounds (Rawat et al., 2021; Rizwanuddin et al., 2023). Additionally, they synthesize diverse organic acids that promote the hydrolysis of inorganic phosphorus minerals (Wei et al., 2018; Kour et al., 2021). PSB also mediate chelation and complexation processes through siderophores and extracellular polysaccharides, thereby increasing soluble phosphate levels (Hamdali et al., 2008; Yi et al., 2008). Through these metabolic activities, PSB can convert 17–34% of fixed soil phosphorus into plant-available forms (Sharma et al., 2013).

Beyond improving soil phosphorus availability, PSB inoculation can modify the composition and abundance of indigenous microbial communities, thereby indirectly enhancing phosphorus solubilization (Pan and Cai, 2023). For example, inoculation with *Pseudomonas asiatica* JP233 significantly altered microbial β-diversity by enriching beneficial taxa associated with plant growth promotion (Tang et al., 2025). Moreover, PSB play an important role in the biogeochemical cycling of phosphorus, with several functional genes identified, including those encoding alkaline phosphatases (*phoD, phoA*), phosphorus uptake and transport (*pst*), and phytases (*phy*) (Dai et al., 2020; Mishra et al., 2024). Collectively, PSB contribute to reducing dependence on chemical phosphorus fertilizers, preventing excessive phosphorus accumulation in soils, regulating soil microbial community composition, preventing nutrient imbalances, and promoting sustainable agricultural productivity. However, most previous studies examining the effects of PSB on plant systems have focused on crops such as maize, wheat, and tomato, while comparatively limited research has examined their influence on cucumber.

*Cucumis sativus* L. (cucumber) is a widely consumed vegetable crop valued for its high vitamin content and notable hypoglycemic and antioxidant properties (Zhang et al., 2021; Khan et al., 2022). China is the world’s largest producer, with an annual output of ~80.2 million tons in 2023 (FAOSTAT), which is primarily attributed to the widespread adoption of facility-based cultivation. However, intensive greenhouse-based vegetable cultivation can lead to declines in soil fertility and disruptions to soil ecological balance, ultimately reducing both yield and nutritional quality (Yan et al., 2024). In particular, phosphorus imbalance severely restricts cucumber growth and substantially decreases productivity (Lee et al., 2024; Sieczko et al., 2024). Therefore, there is an urgent need to identify PSB strains with both effective phosphorus-solubilizing capacity and beneficial rhizosphere microecological regulatory functions for cucumber cultivation.

In this study, a phosphate-solubilizing strain, *B. subtilis* Y31, isolated from cucumber rhizosphere soil, was applied as a microbial inoculant and demonstrated significant growth-promoting effects on cucumber. To elucidate the underlying mechanisms, we integrated rhizosphere soil microbial community profiling with whole-genome sequencing. Rhizosphere microbiome analysis was used to determine how inoculation with strain Y31 alters microbial community composition and diversity. Furthermore, whole-genome sequencing further provided insight into the genetic basis of its phosphorus-solubilizing capacity and plant growth-promoting traits by identifying relevant functional gene clusters. Collectively, our findings establish a scientific basis for the development and agricultural application of strain Y31 and offer new insights into PSB-mediated plant growth promotion from a multi-omics perspective. These results highlight the potential of microbial fertilizer strategies to support sustainable cucumber production.

## 2 Materials and methods

### 2.1 Isolation of strain Y31 and detection of its phosphate solubilizing and growth-promoting abilities

To isolate PSB strains from cucumber rhizosphere soil, a specialized C_6_H_6_Ca_6_O_24_P_6_-based solid medium was used. The medium contained glucose (10.0 g·L^−1^), ammonium sulfate (0.5 g·L^−1^), sodium chloride (0.3 g·L^−1^), potassium chloride (0.3 g·L^−1^), magnesium sulfate heptahydrate (0.3 g·L^−1^), ferrous sulfate heptahydrate (0.03 g·L^−1^), manganese sulfate monohydrate (1.0 g·L^−1^), C₆H₆Ca₆O₂₄P₆ (5.0 g·L^−1^), and agar (15 g·L^−1^), with the pH adjusted to 7.2–7.4. Soil samples (5 g) were suspended in 45 mL of sterile distilled water and shaken at 180 rpm and 37 °C for 30 min, followed by sedimentation for 5 min. Serial dilutions were prepared up to 10^−6^, and 100 μL aliquots from the 10^−3^ to 10^−6^ dilutions were spread onto the solid medium and incubated at 37 °C for 120 h. Colonies producing clear zones were isolated and purified. The isolate with the highest phosphate solubilization index (SI) was selected for subsequent analyses. The SI was calculated as the ratio of the total diameter (colony + halo zone) to the colony diameter (both in mm) (Ibáñez et al., 2021).

Single purified colonies were inoculated into Luria-Bertani (LB) medium (medium composition per liter: 5 g yeast extract, 10 g NaCl, 10 g tryptone) and cultured at 37 °C with shaking (180 rpm) for 12 h. The resulting cultures were transferred to C_6_H_6_Ca_6_O_24_P_6_ liquid medium at a 1% inoculum volume. Samples were collected every 24 h. After centrifugation, available phosphorus in the supernatant was quantified using the molybdenum–antimony colorimetric method to assess phosphate solubilization capacity (Lü et al., 2023), and the pH was measured using a calibrated pH meter. Parallel experiments were conducted to evaluate the Ca_3_(PO_4_)_2_ solubilization capacity of strain Y31 by replacing the phosphorus source under identical culture conditions.

Bacterial culture of strain Y31 was prepared by incubating the strain in LB liquid medium at 37 °C with shaking at 180 rpm for 12 h. Protease activity was assessed using skim milk agar for 48 h at 37 °C (medium composition per liter: 2 g yeast extract, 4 g NaCl, 50 g skim milk, 4 g peptone, and 15 g agar). A 10 μL aliquot of the bacterial suspension was spotted onto skim milk agar, and protease production was indicated by the formation of clear zones surrounding the colonies. Amylase activity was detected using colonies grown for 48 h at 37 °C on starch agar (per liter: 10 g soluble starch, 20 g peptone, 20 g glucose, 10 g yeast extract, and 20 g agar). Lugol’s iodine solution (1% iodine in 2% potassium iodide, w/v) was applied to the plate surface, and amylase activity was identified by the presence of a colorless halo against the dark iodine–starch complex (Xu et al., 2022). Cellulase activity was evaluated using colonies grown for 48 h at 37 °C on carboxymethylcellulose (CMC) agar [per liter: 2 g CMC-Na, 0.5 g MgSO_4_·7H_2_O, 2 g (NH_4_)_2_SO_4_, 1 g K_2_HPO_4_, 0.5 g NaCl, 1% (w/v) Congo Red, and 15 g agar]. Cellulase production was indicated by a clear hydrolysis zone after colony growth (Teather and Wood, 1982). β-mannanase activity was assayed using colonies grown for 3 days at 37 °C on konjac gum agar with the same basal formulation as the CMC agar described above, except replacing CMC with 2 g konjac gum. The presence of clear zones surrounding colonies signified β-mannanase activity. Siderophore production was evaluated using colonies grown for 3 days at 37 °C in the dark on Chrome Azurol S (CAS) blue agar [per liter: 10 mL of 20% sucrose, 30 mL of 10% acid-hydrolyzed casein, 1 mL of 1 mM CaCl_2_, 5 mL of 0.1 M phosphate-buffered saline (pH 6.8), 50 mL of CAS dye solution, and 18 g agar; pH 7.2]. Siderophore activity was indicated by a color shift from blue to orange around the colonies (Schwyn and Neilands, 1987).

To assess the antifungal activity of Y31 against *Botrytis cinerea*, a 5-day-old fungal mycelial plug was centrally placed on potato dextrose agar (PDA). The *B. cinerea* strain used in our experiments was isolated from diseased young cucumber fruits. A 5 μL aliquot of bacterial suspension was spotted 2.0 cm from the fungal inoculum. Plates were incubated at 28 °C for 4 days. The percentage inhibition of fungal growth was calculated as: [(*D*_c_ – *D*_b_)/*D*_c_] × 100, where *D*_c_ represents the fungal colony diameter of the control group (CK), and *D*_b_ denotes the colony diameter of the treated group after application of strain Y31 (Kunfa et al., 2025).

### 2.2 Phylogenetic tree analyses

Genomic DNA of strain Y31 was extracted using the EasyPure^®^ Bacteria Genomic DNA Kit (TransGen Biotech Co., Ltd., Beijing, China). The 16S rDNA gene was amplified using the following primers: forward, 5’-GGATCTTCCAGAGATAGAGTTTGATCCTGGTCAG-3′; and reverse, 5’-CTGCCGTTCGACGATTACGGCTACCTTGTTACGACTTC-3′. The amplified product was sequenced by Personal Biotechnology Co., Ltd. (Shanghai, China).

The 16S rDNA sequence was analyzed using BLAST (NCBI) to identify closely related bacterial taxa. Phylogenetic comparison was performed in MEGA12, and representative homologous strains were selected to construct a neighbor-joining phylogenetic tree. Complete genome sequences of closely related strains were obtained from the NCBI database. Average Nucleotide Identity based on BLAST (ANIb) was calculated using JSpeciesWS (http://jspecies.ribohost.com/jspeciesws/, v5.0.1) (Richter et al., 2016).

### 2.3 Plants growth promotion assays

For preparation of Y31 bacterial agent, spores were induced using a dedicated sporulation medium optimized for preservation stability. The medium composition included (per liter): ammonium sulfate (4 g), yeast extract (4 g), dipotassium phosphate (1 g), magnesium sulfate heptahydrate (0.82 g), manganese chloride (0.08 g), calcium chloride dihydrate (0.16 g), and glucose (2 g). Following initial culture in LB medium, the strain was transferred to a 5 L fermenter (Baoxing Bio-Engineering Equipment Co., Ltd., Shanghai, China) at an inoculation rate of 2%. Fermentation was conducted at 37 °C with agitation at 200 rpm for 72 h. Spores were harvested by centrifugation at 9,000 rpm for 30 min, followed by a two-step purification with sterile water. The purified spores were resuspended in 40 mL sterile water and quantified by colony enumeration on LB agar plates.

To determine the optimal application concentration of the Y31 bacterial agent for cucumber cultivation, a seedling growth experiment was conducted between June and July 2024. Pots with a 13 cm diameter were used. Soil was passed through a 2 mm sieve and mixed with nutrient soil at a 2:1 ratio, with 1 kg of the mixture added to each pot. The experiment consisted of four treatments: a control (CK) and three inoculation treatments (T1, T2, T3), with Y31 bacterial inoculum applied at 8 × 10^9^, 16 × 10^9^, and 24 × 10^9^, CFU/m^2^, respectively. Each treatment was replicated four times, resulting in 16 pots in total. Cucumber seeds (cultivar Jinyou 209; Beijing Wanlongyufeng Seed Co., Ltd., China) were sown directly without pre-treatment, with five seeds per pot. The plants were grown in a culture room at 25 °C, 80 ± 10% relative humidity, and 12,000 Lux light intensity with a photoperiod of 16 h light and 8 h dark. After 30 days, plant height, stem diameter, shoot fresh weight, and shoot dry weight were measured. Treatment performance was compared, and the bacterial concentration that most effectively promoted cucumber growth was selected for subsequent experiments.

For the greenhouse experiment, cucumber seedlings (Jinyou 209) were transplanted into soil at the Shijiazhuang Academy of Agriculture and Forestry Sciences on August 16, 2024. The Y31 suspension at 16 × 10^9^ CFU/m^2^ was applied at transplantation, followed by a second application 15 days later. Cucumber fruits reached harvest maturity approximately 30 days after planting. At this stage, six plants per treatment were randomly selected to assess plant height, stem diameter, and SPAD values. Fruits were harvested every 4 days over multiple harvest cycles. During each harvest, the fruit count and individual fruit weight were recorded. Total yield was defined as the cumulative fruit weight per plant across all harvests.

### 2.4 Characterization of the cucumber rhizosphere microbiome

Rhizosphere soil was collected by gently shaking the roots to remove loosely adhered soil, followed by brushing to obtain tightly adhering rhizosphere soil. A portion of each sample was submitted to the Hebei Academy of Agriculture and Forestry Sciences (Shijiazhuang, China) for physicochemical property analysis. The remaining samples were stored at −80 °C and sent to Pasenuo Biotechnology Co., Ltd. for microbiome sequencing.

Following high-throughput sequencing on the Illumina platform, data quality control, noise filtering, paired-end read assembly, and chimera removal were performed using the DADA2 plugin in QIIME 2019.4. This process yielded high-quality amplicon sequence variants (ASVs). Redundant sequences were subsequently removed via 100% similarity clustering to generate an ASV abundance matrix. Microbial diversity analyses were conducted in QIIME, incorporating α-diversity metrics (Chao1 index for species richness and Shannon index for community evenness) and β-diversity assessments based on Bray–Curtis dissimilarity to compare community composition among samples.

### 2.5 Bacterial genome assembly and functional annotation

For whole-genome sequencing, strain Y31 was inoculated into LB medium and incubated overnight at 37 °C with shaking at 180 rpm. Cells were harvested by centrifugation at 5,000 rpm for 10 min at 4 °C. Second- and third-generation sequencing were subsequently performed by Personal Biotechnology Co., Ltd. Genome assembly of the third-generation sequencing data was carried out using Unicycler, Flye, Hifiasm, and Necat. The resulting assemblies were polished with Pilon, using high-quality second-generation sequencing reads for error correction. This integrated assembly strategy produced a complete genome sequence that met stringent quality criteria.

Protein-coding genes in the Y31 genome were predicted using GeneMarkS (v4.32). tRNA genes were identified with tRNAscan-SE, and rRNA genes were annotated using Barrnap. Non-coding RNAs were further annotated by comparison with the Rfam database. CRISPR-associated repeats and spacers were detected using CRISPRCasFinder, while potential prophage regions and genomic islands were identified using PhiSpy and IslandViewer 4, respectively. Functional annotation was performed through alignment against multiple databases, including Swiss-Prot, KEGG (Kyoto Encyclopedia of Genes and Genomes), COG (Clusters of Orthologous Groups), NR (Non-Redundant Protein Database), CARD (Comprehensive Antibiotic Resistance Database), CAZy (Carbohydrate-Active enZYmes Database) (Drula et al., 2021), and antiSMASH (antibiotics and Secondary Metabolite Analysis Shell) (Blin et al., 2025). The draft genome sequence has been deposited in the NCBI database under BioProject accession PRJNA1288737 (BioSample accession SAMN42432703).

### 2.6 Statistical analyses

Statistical comparisons between the CK and Y31 groups were conducted using one-way ANOVA in IBM SPSS Statistics 20.0. Data visualizations, including bar plots, Venn diagrams, and species distribution plots, were generated using Origin 2024. Principal coordinates analysis (PCoA) was performed with the vegan package in R 4.4.2.

## 3 Results

### 3.1 Phosphate-solubilizing and growth-promoting ability of strain Y31

**Figure 1:**
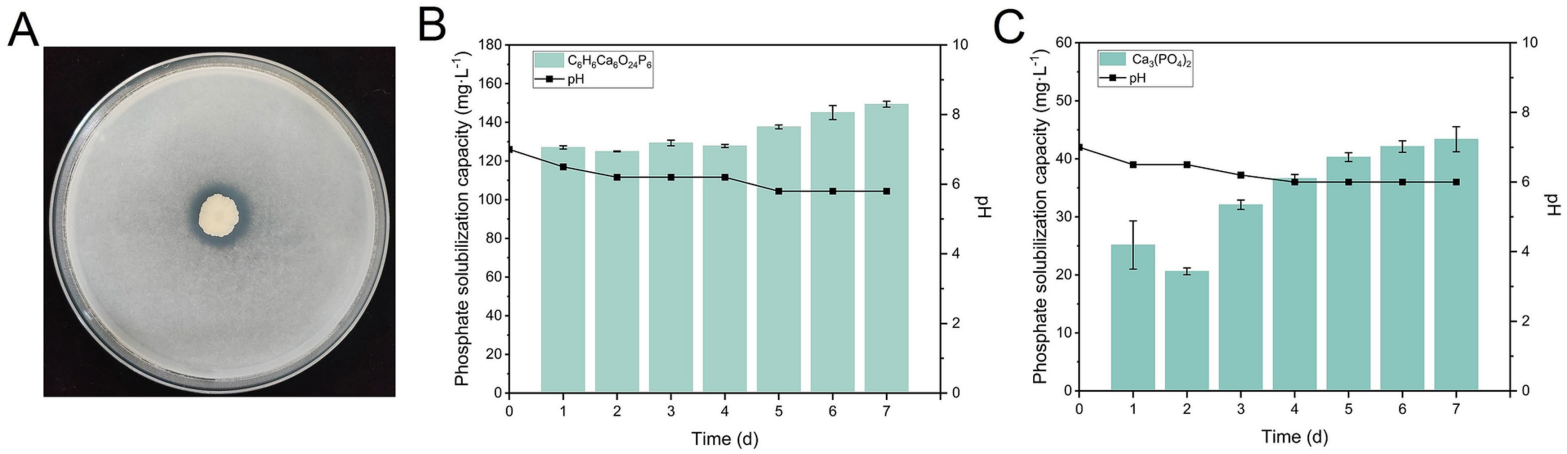
Screening of strain Y31 for phosphate-solubilizing activity. **(A)** Formation of a clear phosphate-solubilization halo by strain Y31 on calcium phytate agar plates. **(B)** Time-dependent changes in available phosphorus concentration in C_6_H_6_Ca_6_O_24_P_6_ liquid medium over a 7-day incubation period. **(C)** Time-dependent changes in available phosphorus concentration in Ca_3_(PO_4_)_2_ liquid medium during a 7-day culture period.

Strain Y31 was isolated from cucumber rhizosphere soil using calcium phytate (C_6_H_6_Ca_6_O_24_P_6_) agar as the selective screening medium. Distinct halo zones formed around Y31 colonies on plates containing calcium phytate, and the SI of 1.55 ± 0.01 was recorded on day 7 (Figure 1A), demonstrating effective organic phosphate solubilization. To further assess its phosphate-solubilizing capacity, calcium phytate and tricalcium phosphate were separately added to liquid culture media. Quantitative analysis showed that dissolved phosphorus released from calcium phytate remained stable at 127.28 mg·L^−1^ during the first 96 h, followed by a gradual increase to a maximum of 149.37 mg·L^−1^ by day 7 (Figure 1B). Concurrent pH measurements revealed a gradual acidification of the culture supernatant from pH 7.0 to 5.8 by day 5, after which the pH stabilized. Strain Y31 also solubilized tricalcium phosphate, reaching 43.38 mg·L^−1^ of soluble phosphorus along with a corresponding pH decrease from 7.0 to 6.0 (Figure 1C).

**Figure 2:**
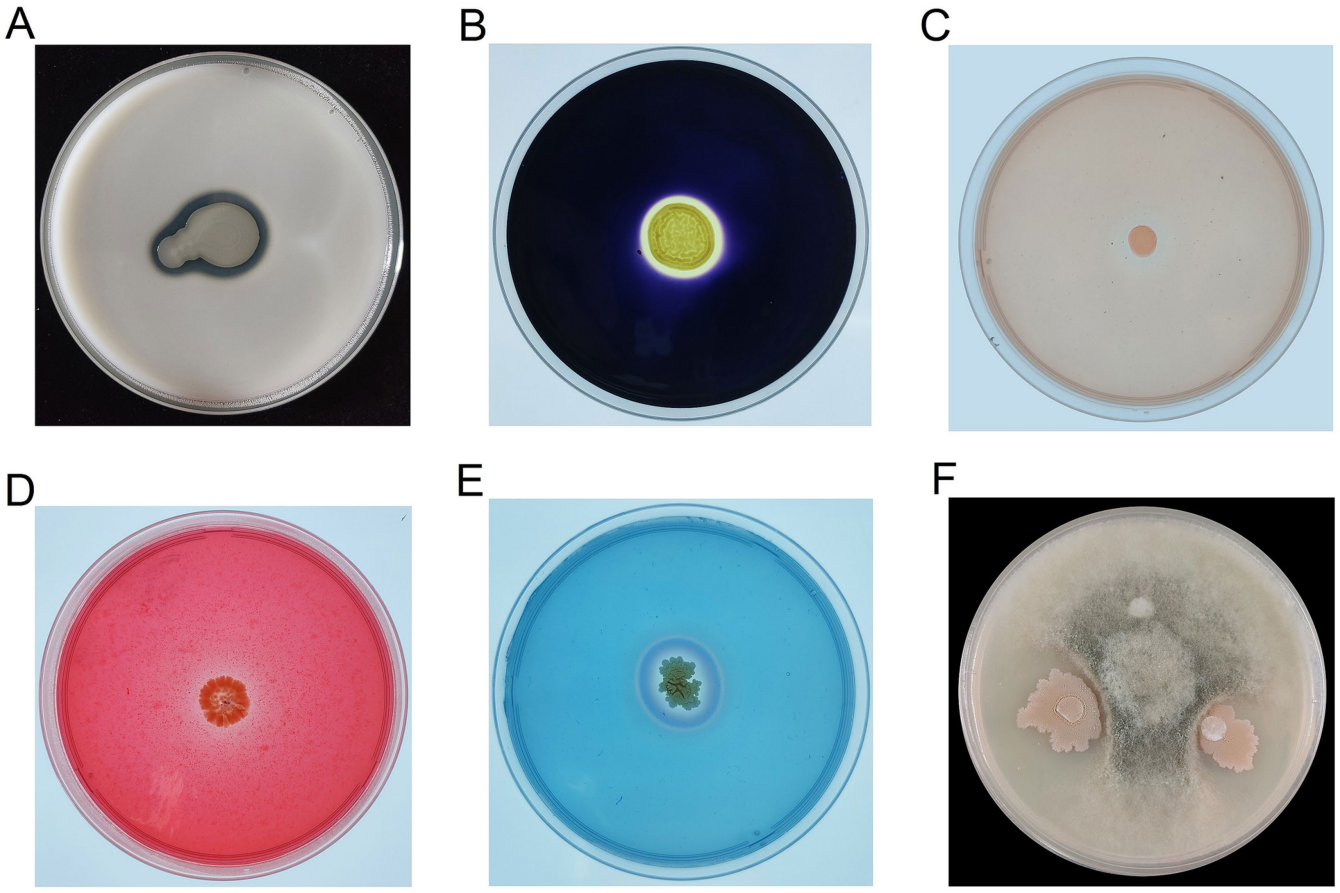
Growth-promoting and antifungal activities of strain Y31. **(A)** Protease production; **(B)** amylase production; **(C)** cellulase production; **(D)** β-mannanase production; **(E)** siderophore production; **(F)** antagonistic activity against *B. cinerea*.

In addition to phosphate solubilization, Y31 exhibited multiple plant growth-promoting traits. It produced extracellular protease (Figure 2A), amylase (Figure 2B), cellulase (Figure 2C), and β-mannanase (Figure 2D), and was capable of siderophore synthesis (Figure 2E). Furthermore, Y31 also demonstrated biocontrol potential, significantly inhibiting the mycelial growth of *B. cinerea* with a suppression rate of 66.29 ± 1.70% (Figure 2F).

### 3.2 Identification of strain Y31

**Figure 3:**
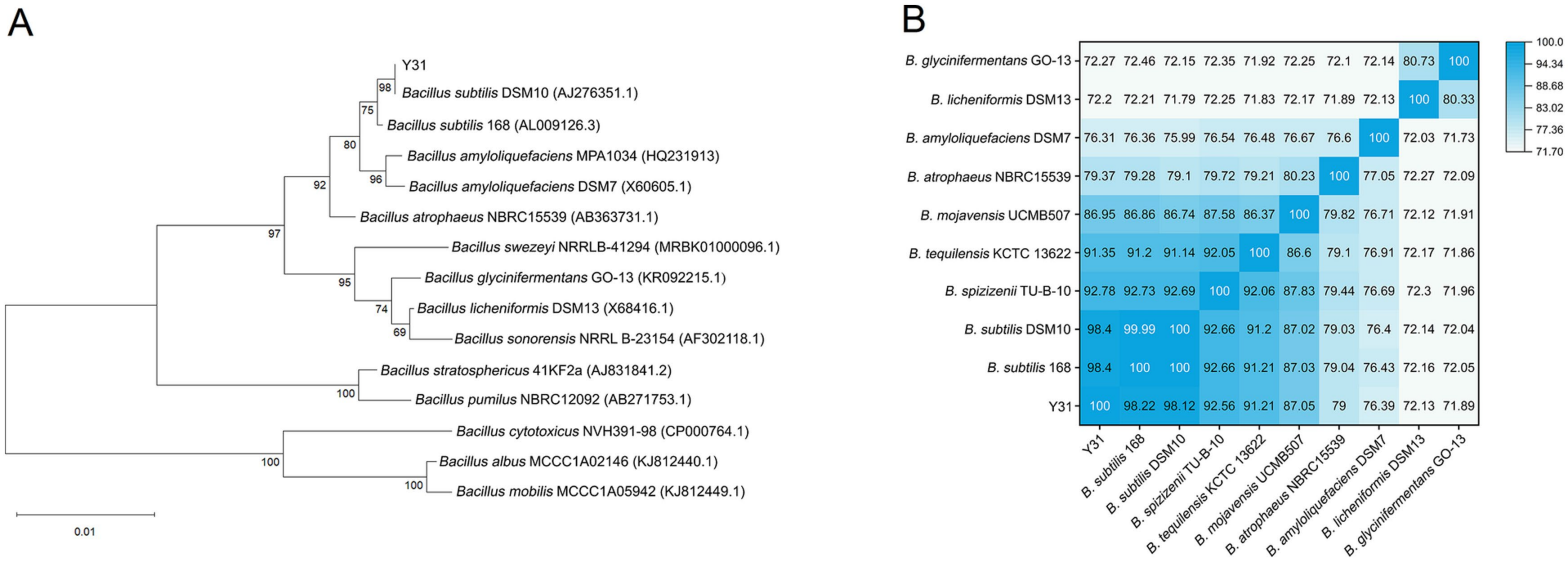
Identification of strain Y31. **(A)** Phylogenetic analysis of strain Y31 based on 16S rDNA sequences; **(B)** ANIb comparison of strain Y31 with nine other *Bacillus* species.

Phylogenetic analysis based on the 16S rDNA sequence identified Y31 as belonging to the *B. subtilis* species, clustering with *B. subtilis* DSM10 and *B. subtilis* 168 (Figure 3A). Whole-genome comparisons further supported this classification, as Y31 exhibited ANIb values of 98.4% with both reference strains, whereas ANIb values with seven other *Bacillus* species ranged from 71.89 to 92.78% (Figure 3B). Collectively, these findings supported the classification of strain Y31 as *B. subtilis*.

### 3.3 Growth-promoting effects of *B. subtilis* Y31 on cucumber plants

**Figure 4:**
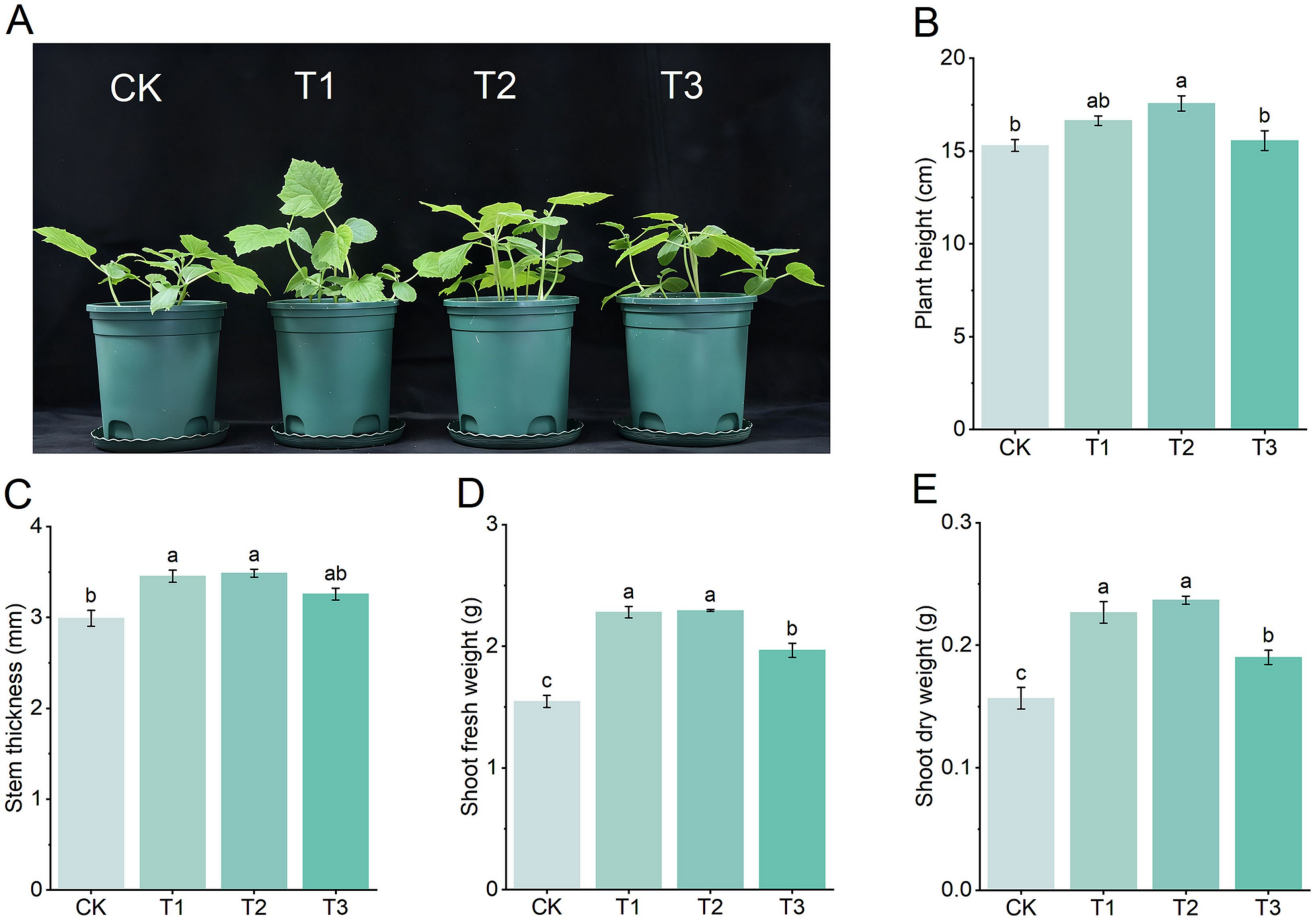
Effects of different concentrations of strain Y31 on the growth of potted cucumber plants. **(A)** Cucumber seedlings inoculated with strain Y31 at 30 dpi. CK, T1, T2, and T3 were treated with Y31 at concentrations of 0, 8, 16, and 24 × 10^9^ CFU/m^2^, respectively. **(B)** Plant height, **(C)** stem thickness, **(D)** shoot fresh weight, and **(E)** shoot dry weight at 30 dpi. Bars represent the mean ± SEM of three independent biological replicates (*n* = 5). Different lowercase letters (a, b) indicate statistically significant differences according to Tukey’s test (*p* < 0.05). The letters a and b indicate significant differences between two samples at *p* < 0.05.

To evaluate the effects of *B. subtilis* Y31 application on cucumber growth and yield, both seedling growth experiments and greenhouse cultivation trials were conducted. In the seedling growth experiment, strain Y31 was applied at three concentrations: 8, 16, and 24 × 10^9^ CFU/m^2^ (Figure 4A). At 30 days post-inoculation (dpi), seedlings treated with Y31 exhibited significantly greater plant height (Figure 4B), stem diameter (Figure 4C), shoot fresh weight (Figure 4D), and shoot dry weight (Figure 4E) than those in the CK treatment. The most pronounced growth promotion occurred at 16 × 10^9^ CFU/m^2^, where plant height increased by 14.81%, stem diameter by 16.61%, shoot fresh weight by 48.28%, and shoot dry weight by 51.06% relative to CK. Therefore, this concentration was selected for the subsequent greenhouse experiments.

**Figure 5:**
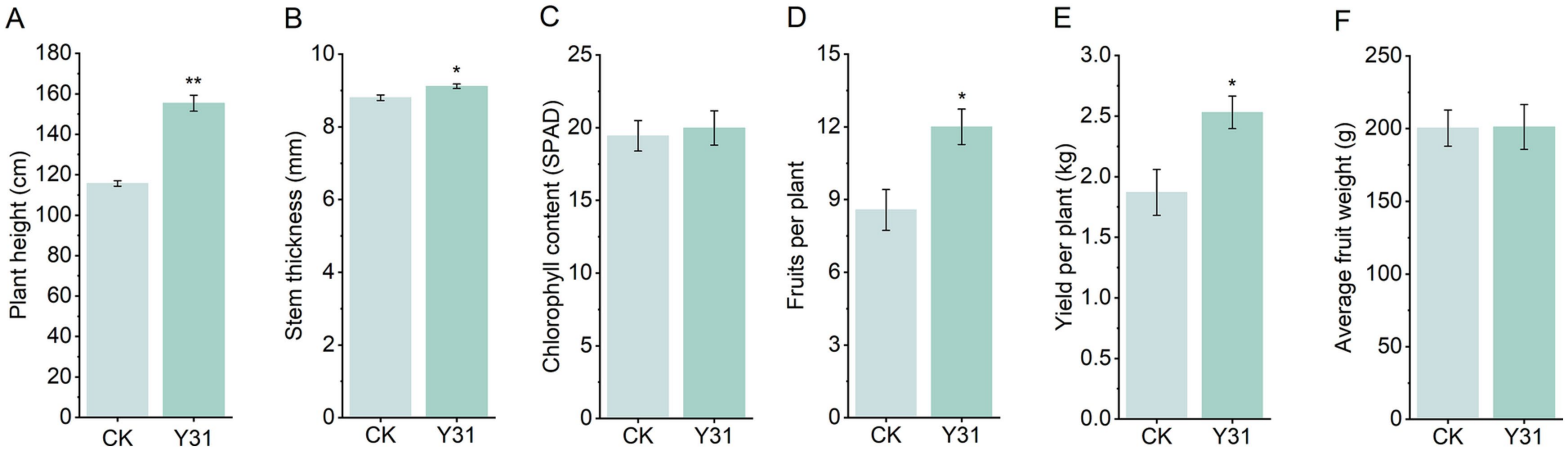
Effects of strain Y31 on cucumber growth and productivity in a greenhouse experiment. **(A)** Plant height, **(B)** stem thickness, and **(C)** chlorophyll content (SPAD) measured at 30 dpi; **(D)** number of fruits per plant, **(E)** fruit yield per plant, and **(F)** average fruit weight recorded at harvest. *, ** Indicate statistically significant differences relative to CK at *p* < 0.05 and *p* < 0.01, respectively.

To further assess the growth-promoting effects of Y31, a controlled greenhouse trial was performed. At 30 dpi, Y31-treated plants again showed significantly increased plant height and stem diameter compared to CK (Figures 5A,B). Notably, Y31 application markedly enhanced productivity, with each plant producing an average of 12.00 marketable fruits and a total yield of 2.53 kg per plant—representing increases of 40.02 and 35.30%, respectively, over CK (Figures 5D,E). However, no significant differences were detected in chlorophyll content or individual fruit weight between treatments (Figures 5C,F). Overall, these findings demonstrated that Y31 application effectively promoted cucumber growth and significantly improved yield under greenhouse cultivation.

In addition, the effects of Y31 inoculation on rhizosphere soil physicochemical properties were examined in the greenhouse-grown plants. Inoculation with Y31 significantly enhanced the availability of key soil nutrients (Table 1). Organic matter (OM) content increased by 11.77% relative to CK, and available phosphorus (AP) increased by 16.33%, accompanied by an improved AP-to-total phosphorus (TP) ratio. Total soil nitrogen (TN) and ammonium nitrogen (AN) increased by 3.05 and 5.93%, respectively, whereas no significant differences were observed in total potassium (TK) or available potassium (AK). A slight reduction in soil pH was also observed (from 7.80 ± 0.00 to 7.76 ± 0.01). These findings indicated that Y31 inoculation improved soil fertility in greenhouse cucumber production systems, primarily through enhanced phosphorus solubilization and associated improvements in soil nutrient availability and physicochemical properties.

**Table 1:** Soil properties of CK and Y31 treatment.

Properties	CK	*B. subtilis* Y31
OM (g·kg^−1^)	27.95 ± 0.05	31.24 ± 0.16^***^
TP (g·kg^−1^)	2.12 ± 0.00	2.26 ± 0.01^***^
AP (mg·kg^−1^)	73.73 ± 0.27	85.77 ± 0.41^***^
AP/TP (%)	3.58 ± 0.01	3.79 ± 0.03^***^
TN (g·kg^−1^)	1.31 ± 0.01	1.35 ± 0.00^**^
AN (mg·kg^−1^)	84.47 ± 0.51	89.48 ± 0.62^**^
TK (g·kg^−1^)	18.25 ± 0.02	18.12 ± 0.08
AK (mg·kg^−1^)	291.33 ± 1.86	284.00 ± 2.08
pH	7.80 ± 0.00	7.76 ± 0.01^**^

OM, organic matter; TP, total phosphorus; AP, available phosphorus; TN, total nitrogen; AN, available nitrogen; TK, total potassium; AK, available potassium. ^**^, ^***^ indicated statistically significant differences relative to CK at *p* < 0.01 and *p* < 0.001, respectively.

### 3.4 Soil rhizosphere microbiome dynamics influenced by *B. subtilis* Y31

To evaluate the effects of strain Y31 on the cucumber rhizosphere environment, microbial biodiversity and community structure were assessed. A total of 721,105 high-quality V3–V4 bacterial 16S rRNA gene sequences and 795,961 ITS3–4 fungal sequences were obtained across all samples, with average read lengths of 418 bp and 243 bp, respectively. The dilution curves for both bacterial and fungal datasets reached clear saturation (Supplementary Figure 1), indicating sufficient sequencing depth for comprehensive community characterization.

**Figure 6:**
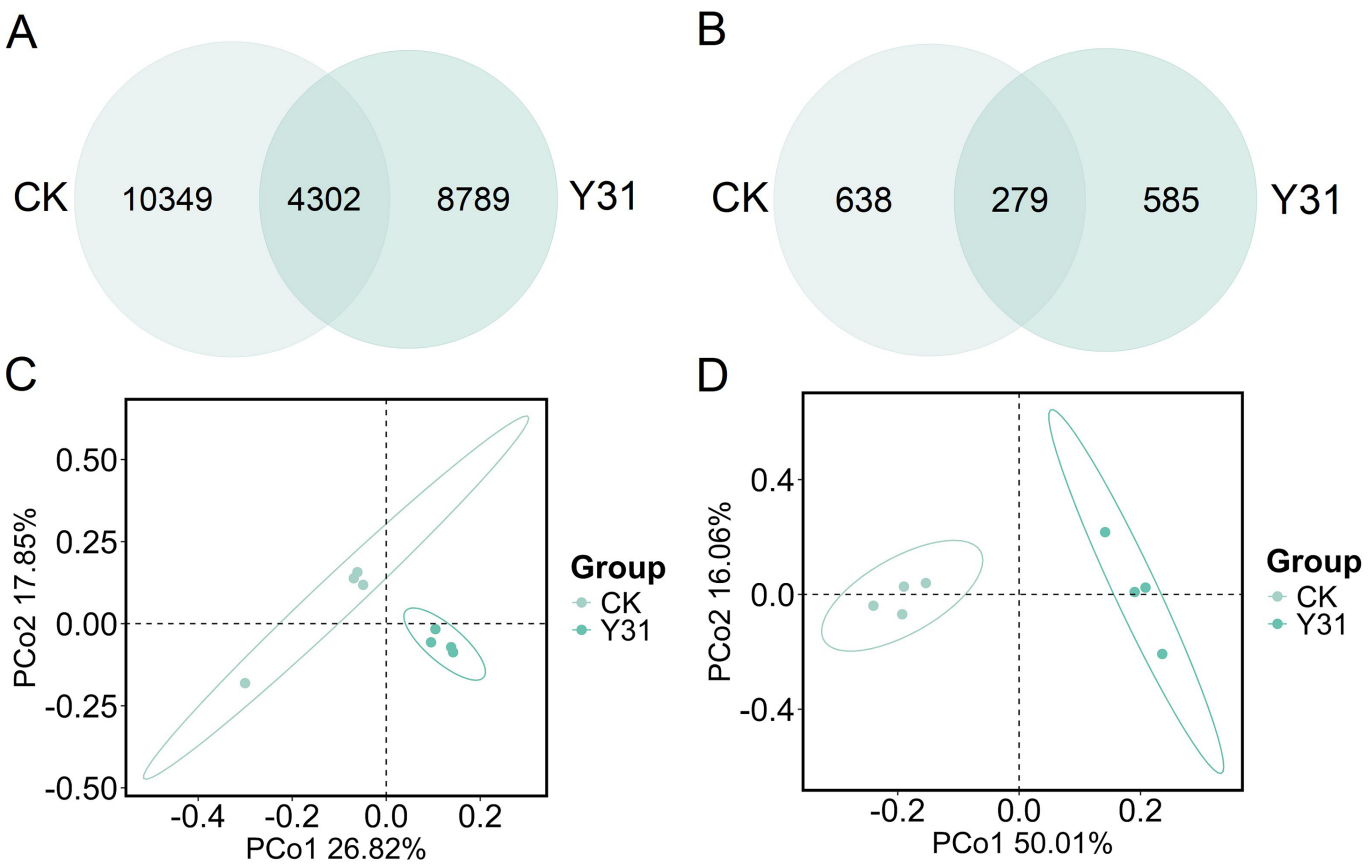
Microbial community characteristics and beta diversity index in rhizosphere soils under CK and Y31 treatments. **(A)** Distribution of bacterial OTUs (Venn diagram). **(B)** Distribution of fungal OTUs (Venn diagram). **(C)** PCoA of bacterial communities. **(D)** PCoA of fungal communities.

Venn diagram analysis further illustrated the distribution of shared and unique operational taxonomic units (OTUs) between treatments. In total, 23,440 bacterial OTUs were identified, of which 10,439 were unique to the CK group and 8,789 were exclusive to the Y31-treated group (Figure 6A). Fungal community profiling revealed 1,502 total OTUs, with 638 and 585 uniquely associated with CK and Y31-treated samples, respectively (Figure 6B). These results indicate that Y31 application reduced the number of treatment-specific microbial taxa within both bacterial and fungal assemblages relative to the control.

Alpha-diversity indices were subsequently evaluated to characterize microbial richness and diversity in the rhizosphere. For bacterial communities, the Chao1, observed species, Shannon, and Simpson indices did not significantly differ between CK and Y31 treatments (Table 2). In contrast, fungal communities exhibited significant reductions in Chao1 and observed species indices following Y31 application (Table 2). These findings suggested that Y31 treatment maintained bacterial community richness and diversity, while significantly decreasing fungal richness in the cucumber rhizosphere.

**Table 2:** Microbial alpha diversity index in the soils of CK and Y31 treatments.

Kingdom	Treatments	Chao1	Observed species	Shannon	Simpson
Bacteria	CK	5466.84 ± 133.34	5398.75 ± 137.58	11.19 ± 0.10	0.999 ± 0.00
	Y31	5152.04 ± 212.55	5067.80 ± 233.46	11.08 ± 0.05	0.999 ± 0.00
Fungi	CK	366.36 ± 7.15	364.50 ± 6.86	5.54 ± 0.06	0.946 ± 0.00
	Y31	328.01 ± 10.39^*^	327.20 ± 10.36^*^	5.52 ± 0.14	0.950 ± 0.01

*Indicated statistically significant differences relative to CK at *p* < 0.05.

To further examine Y31 treatment-driven shifts in community structure, beta-diversity analysis was performed using Bray–Curtis dissimilarity. PCoA indicated clear separation between CK and Y31-treated samples. For bacterial communities, the first two coordinate axes explained 26.82 and 17.85% of the variance, respectively (Figure 6C). Fungal communities showed even stronger differentiation, with the first two axes accounting for 50.01 and 16.06% of the variance (Figure 6D). These ordination patterns demonstrate that Y31 application altered both bacterial and fungal community structures, with more pronounced compositional shifts observed in fungal assemblages, indicating potential impacts on rhizosphere ecological dynamics.

**Figure 7:**
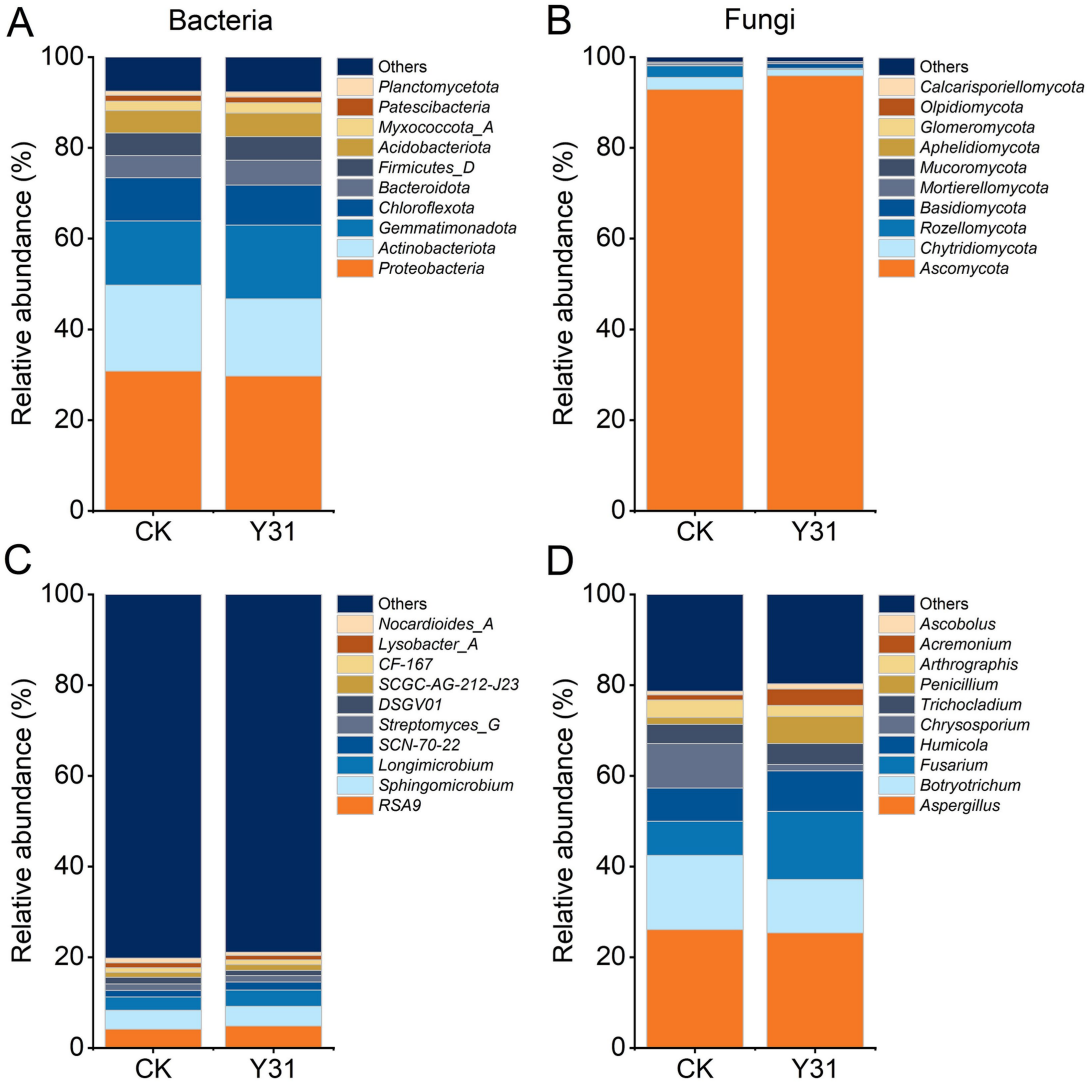
Taxonomic composition of rhizosphere soil microorganisms at the phylum and genus levels under CK and Y31 treatments. **(A)** Bacterial composition at the phylum level; **(B)** fungal composition at the phylum level; **(C)** bacterial composition at the genus level; **(D)** fungal composition at the genus level.

To further characterize structural variations in microbial communities, taxonomic profiles of both bacterial and fungal populations were examined. In cucumber rhizosphere soils, the predominant bacterial phyla—*Proteobacteria*, *Actinobacteriota*, *Gemmatimonadota*, *Chloroflexota*, *Bacteroidota*, *Firmicutes_D*, *Acidobacteriota*, *Myxococcota_A*, *Patescibacteria*, and *Planctomycetota*—collectively accounted for 91.81–93.59% of total bacterial sequences (Figure 7A). Notably, the relative abundance of *Actinobacteriota* was reduced by 10.38% following Y31 application compared with the CK group (Table 3). Fungal communities were dominated by *Ascomycota*, comprising 87.33–96.77% of total fungal sequences (Figure 7B). Relative to the control, Y31 application significantly decreased the relative abundance of *Rozellomycota* and *Mucoromycota*, while promoting an increase in *Basidiomycota* (Table 3).

**Table 3:** Relative abundance of differential species at the phylum and genus levels.

Level	Kingdom	Species	CK	*B. subtilis* Y31
Phylum	Bacteria	*Actinobacteriota*	19.00 ± 0.34	17.03 ± 0.31^**^
	Fungi	*Rozellomycota*	2.58 ± 0.35	0.25 ± 0.02^***^
		*Basidiomycota*	0.18 ± 0.03	0.59 ± 0.09^**^
		*Mucoromycota*	0.25 ± 0.06	0.01 ± 0.01^**^
Genus	Bacteria	*DSGV01*	1.44 ± 0.02	1.14 ± 0.04^***^
		*Nocardioides*	1.05 ± 0.04	0.73 ± 0.05^**^
	Fungi	*Botryotrichum*	16.38 ± 0.45	11.73 ± 0.69^**^
		*Chrysosporium*	9.80 ± 1.14	1.39 ± 0.22^***^
		*Penicillium*	1.55 ± 0.30	5.97 ± 0.63^***^

**, ***Indicated statistically significant differences relative to CK at *p* < 0.01 and *p* < 0.001, respectively.

At the genus level, bacterial communities were primarily composed of *RSA9*, *Sphingomicrobium*, *Longimicrobium*, *SCN-70-22*, and *Streptomyces_G* (Figure 7C). Y31 treatment significantly reduced the relative abundance of *DSGV01* and *Nocardioides* compared to the control (Table 3). Fungal community profiles were dominated by *Aspergillus*, *Botryotrichum*, *Fusarium*, *Humicola*, *Chrysosporium*, *Trichocladium*, *Penicillium*, *Arthrographis*, *Acremonium*, and *Ascobolus* (Figure 7D). Comparative analysis indicated that Y31 application markedly suppressed *Botryotrichum* and *Chrysosporium*, while enhancing the relative abundance of *Penicillium* (Table 3).

Overall, these results indicate that Y31 application had a relatively limited effect on bacterial community composition but induced pronounced shifts in fungal communities.

### 3.5 Genomic analysis of *B. subtilis* Y31

**Figure 8:**
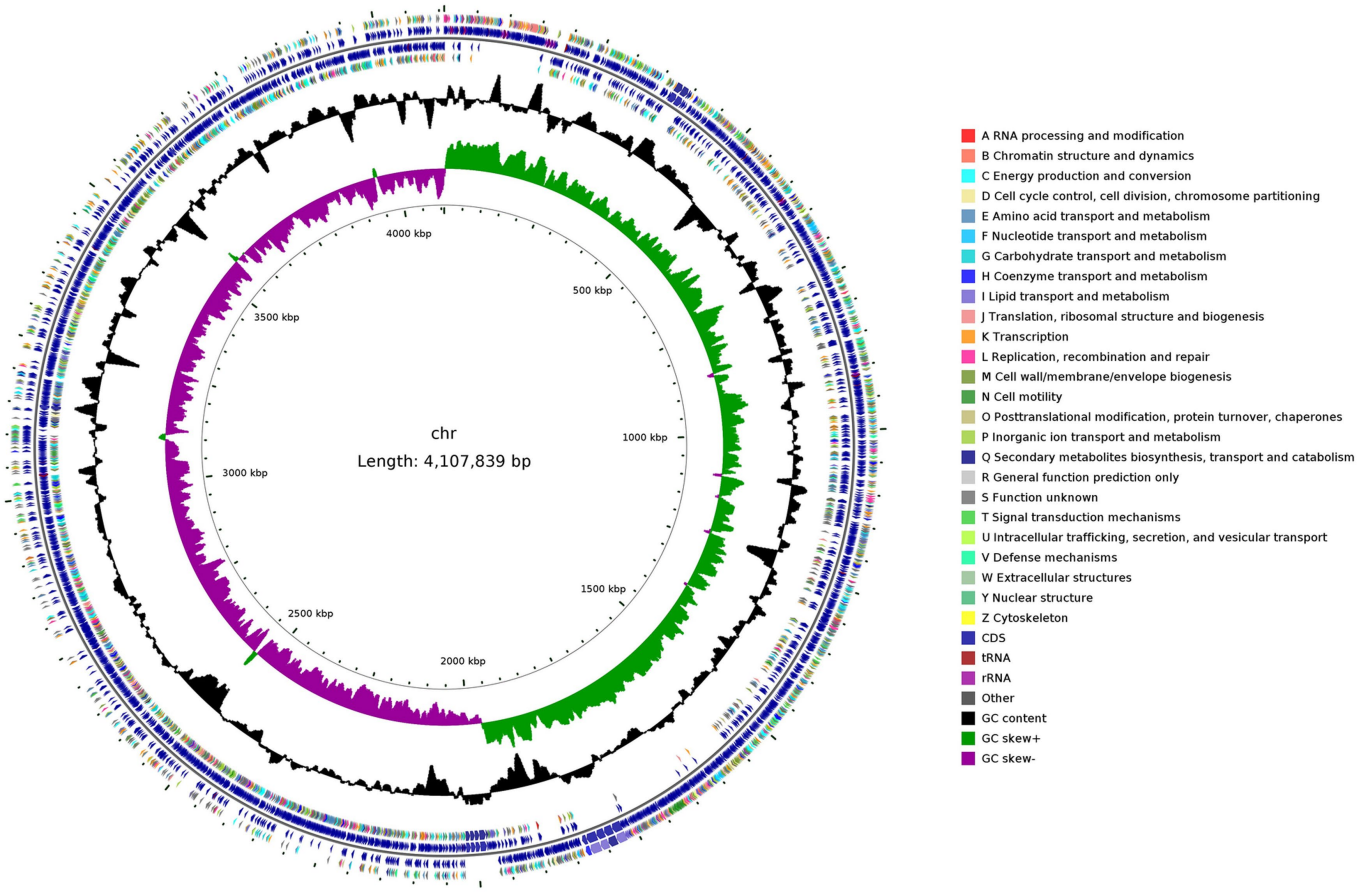
Genomic features of strain Y31. The circular genome map shows concentric layers from center to edge: scale markers, GC skew, GC content, COG functional annotations (layers four and seven), and the genomic positions of protein-coding genes, tRNAs, and rRNAs (layers five and six).

To further elucidate the molecular basis of Y31-mediated plant growth promotion, whole-genome sequencing was conducted. The genome of Y31 consisted of a circular chromosome measuring 4,107,839 bp, with an average GC content of 43.78% (Table 4). A total of 4,161 open reading frames (ORFs) were predicted, representing 87.96% of the genome and covering 3,613,173 bp, with a slightly elevated GC content (44.51%) in coding regions. Among these ORFs, 261 contained signal peptide sequences, 1,148 encoded at least one transmembrane helix, 148 were predicted to encode secreted proteins, and 884 were associated with membrane transport functions. The genome also contained 30 rRNA genes, 86 tRNA genes, 91 noncoding RNA elements, 2 CRISPR arrays, 5 prophage insertions, and 16 genomic islands (Table 4 and Figure 8). Functional annotation across multiple databases revealed that 4,143 genes (99.57%) were assigned in the NR database, 3,831 (92.07%) in Swiss-Prot, 3,905 (93.85%) in COG, and 2,273 (54.63%) in KEGG.

**Table 4:** General features of strain Y31 genome.

Attributes	*B. subtilis* Y31
Genome size	4,107,839 bp
GC content	43.78%
ORF number	4,161
ORF total length	3,613,173 bp
ORF/Genome	87.96%
GC content in ORF region	44.51%
rRNA	10,10,10 (5 s, 16 s, 23 s)
tRNA	86
ncRNA	91
CRISPR Array	2
Prophage	5
Genomics Islands	16

ORF, open reading frame; rRNA, ribosomal ribonucleic acid; tRNA, transfer ribonucleic acid; ncRNA, non-coding ribonucleic acid; CRISPR Array, clustered regularly interspaced short palindromic repeats array.

Furthermore, genomic analysis of strain Y31 identified multiple genes associated with phosphate metabolism, including those encoding alkaline phosphatases (*phoA*, *phoB*, and *phoD*), the two-component regulatory system for phosphatase activity (*phoR*–*phoP*), phytase (*phy*), phosphate-binding transport protein (*pstS*), ATP-binding phosphate import proteins (*pstB1* and *pstB2*), and the inorganic phosphate transporter (*pit*) (Table 5). The genome also contained the *nasABCDEF* gene cluster involved in nitrite transport and reduction, as well as the *ktrABCD* potassium uptake system. Additionally, numerous genes associated with chemotaxis, flagellar assembly, and motility were detected, including *cheABCDRWY, motAB, flgBCG, fliEFGHIJKLMPQRYZ*, and *flhABF* (Supplementary Table 1).

**Table 5:** Genes related to phosphate metabolism.

Gene symbol	Location	SWISS ID	SWISS description
*phoD*	279,663–281,414	P42251	Alkaline phosphatase D
*phoB*	597,734–599,122	P19405	Alkaline phosphatase 3
*phoA*	980,970–982,346	P19406	Alkaline phosphatase 4
*pit*	1,326,201–1,327,202	034436	Probable low-affinity inorganic phosphate transporter
*phy*	2,157,253–2,158,401	P42094	Phytase
*pstS*	2,451,357–2,452,259	P46338	Phosphate-binding protein PstS
*pstB1*	2,447,852–2,448,634	P46342	Phosphate import ATP-binding protein PstB 1
*pstB2*	2,448,645–2,449,454	P46341	Phosphate import ATP-binding protein PstB 2
*phoR*	2,856,234–2,857,958	P23545	Alkaline phosphatase synthesis sensor protein PhoR
*phoP*	2,857,966–2,858,688	P13792	Alkaline phosphatase synthesis transcriptional regulatory protein

**Figure 9:**
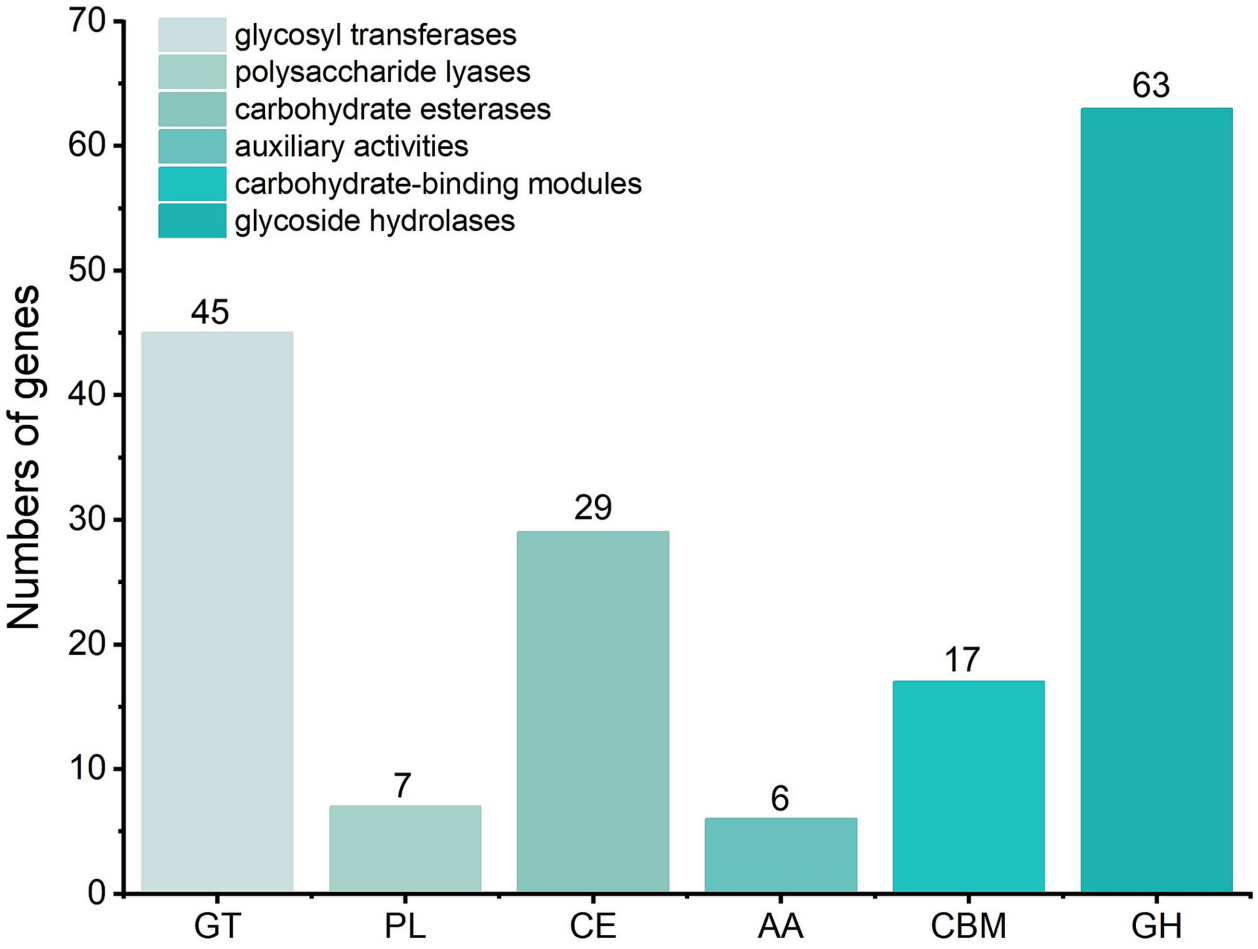
Predictive analysis of carbohydrate-active enzymes within the Y31 genome was conducted through the CAZy database platform.

A CAZy-based genomic survey further revealed 167 carbohydrate-active enzymes encoded within the Y31 genome (Figure 9). These included 63 glycoside hydrolases (GHs), 45 glycosyl transferases (GTs), 29 carbohydrate esterases (CEs), 17 carbohydrate-binding modules (CBMs), 7 polysaccharide lyases (PLs), and 6 auxiliary activity (AA) enzymes. The GH families encompassed enzymes involved in the degradation of diverse plant- and microbe-derived polysaccharides, including lysozymes (GH23, GH73), chitinase (GH18), xylanase (GH11), amylase (GH126), mannanases (GH26, GH76), and glucosidases (GH1, GH3, and GH4).

To assess the biocontrol potential of *B. subtilis* Y31, secondary metabolite biosynthetic gene clusters were examined using the antiSMASH platform. Ten distinct metabolite clusters were identified, eight of which exhibited high similarity to previously characterized clusters (Table 6). Six clusters associated with bacillaene, fengycin, bacillibactin, pulcherriminic acid, subtilosin A, and bacilysin demonstrated 100% sequence identity, while the surfactin biosynthetic cluster showed 78% similarity, and the 1-carbapen-2-em-3-carboxylic acid cluster displayed 16% similarity.

**Table 6:** The secondary metabolism gene clusters in the *B. subtilis* Y31 genome identified using AntiSMASH.

Region	Type	Location	Most similar known cluster	Similarity
Region 1	NRPS	352,655–418,046	Surfactin	78%
Region 2	Terpene	1,114,599–1,135,402	Unknown	ND
Region 3	transAT-PKS, PKS-like, T3PKS, NRPS	1,739,357–1,854,106	Bacillaene	100%
Region 4	NRPS, beta-lactone	1,960,193–2,037,956	Fengycin	100%
Region 5	Terpene	2,099,322–2,121,220	Unknown	ND
Region 6	T3PKS	2,168,972–2,210,069	1-carbapen-2-em-3-carboxylic acid	16%
Region 7	NRP-metallophore, NRPS	3,122,193–3,173,970	Bacillibactin	100%
Region 8	CDPS	3,466,476–3,487,222	Pulcherriminic acid	100%
Region 9	Sactipeptide	3,713,587–3,735,198	Subtilosin A	100%
Region 10	Other	3,741,961–3,783,379	Bacilysin	100%

NRPS, non-ribosomal peptide synthetase; transAT-PKS, trans-acyltransferase-polyketide synthase; PKS-like, polyketide synthase-like; T3PKS, type 3 polyketide synthase; NRP-metallophore, non-ribosomal peptide-metallophore; CDPS, cyclodipeptide synthase; sactipeptide, sulfur-alpha-carbon thioether-containing peptide.

## 4 Discussion

PSB enhance soil fertility by converting insoluble phosphorus compounds into plant-accessible forms, thereby increasing phosphorus availability and supporting crop growth (Timofeeva et al., 2022; García-Berumen et al., 2024). Therefore, the isolation and characterization of new PSB strains are crucial for sustainable agricultural development. Cucumber, a widely cultivated vegetable of high economic value, is frequently grown under protected cultivation systems. However, research on PSB-mediated enhancement of cucumber growth and yield in such environments remains limited. In this study, we isolated a novel PSB strain, *B. subtilis* Y31, from cucumber rhizosphere soil. Inoculation with strain Y31 significantly promoted cucumber growth and yield. By integrating soil microbial community analysis with genomic data, we further elucidated the mechanisms underlying phosphorus solubilization and plant growth promotion by strain Y31.

### 4.1 Phosphate-solubilizing capacity of strain Y31

The Y31 strain demonstrated the capacity to solubilize both organic and inorganic phosphorus (Figure 1). Moreover, inoculation with Y31 improved soil phosphorus activation during cucumber cultivation. Compared to the control treatment (CK), Y31 significantly increased available phosphorus content and phosphorus conversion efficiency (Table 1). Genomic analysis revealed multiple genes associated with phosphorus metabolism and transport (Table 5). The *phoA* and *phoD* genes encode alkaline phosphatases that convert organic phosphorus into inorganic forms (Ragot et al., 2015). In *B. subtilis*, the *phoR–phoP* two-component regulatory system maintains phosphate homeostasis through phosphorylation cascades that activate downstream genes, including *phoA*, *phoB*, and *phoD*, under phosphate-limiting conditions (Santos-Beneit, 2015). Additionally, the *phy* gene encoding phytase, an enzyme responsible for degrading phytate into plant-available phosphate (Singh and Satyanarayana, 2011), was identified in the genome of strain Y31, suggesting its organic phosphate solubilizing capability. The genome also contained high-affinity (*pst*) and low-affinity (*pit*) phosphate transport systems, suggesting dynamic regulation of phosphate uptake (Rao and Torriani, 1990). Genes from these clusters were detected in the genomes of most PSB strains and are closely linked to their plant growth-promoting capabilities (Alaylar et al., 2020; Jeong et al., 2018; Mishra et al., 2024; Xu et al., 2022). Collectively, these genes are central to phosphorus assimilation, transport, and regulatory pathways, enabling Y31 to enhance phosphorus bioavailability in soil ecosystems while maintaining intracellular phosphate homeostasis. Nevertheless, further investigation is needed to elucidate how strain Y31 affects the functional gene composition of the rhizosphere microbial community. Metagenomic analyses focusing on genes involved in soil phosphorus cycling would provide deeper insight into the ecological contributions of Y31 to phosphorus availability.

Furthermore, strain Y31 exhibited a stronger ability to solubilize calcium phytate than tricalcium phosphate (Figure 1), likely reflecting differences in its solubilization mechanisms. Organic phosphorus solubilization appears to be facilitated through the secretion of alkaline phosphatase and phytase (Pang et al., 2024), consistent with the genetic profile of Y31. In contrast, inorganic phosphorus solubilization is primarily mediated by the secretion of organic acids, such as malic, fumaric, citric, and oxalic acids (Wei et al., 2018). For example, *Enterobacter* sp. strain 15S has been reported to produce citric, fumaric, α-ketoglutaric, malic, and oxalic acids, enabling tricalcium phosphate solubilization and enhancement of cucumber root biomass (Zuluaga et al., 2023). Our results demonstrated a concurrent decrease in pH in both the culture solution and soil following inoculation with strain Y31, suggesting that Y31 may release organic acids that facilitate the solubilization of inorganic phosphorus. However, the specific types of organic acids responsible for this acidification require further characterization.

### 4.2 Identification of strain Y31 as *B. subtilis*

Phylogenetic analysis revealed that strain Y31 was classified within the *B. subtilis* group and clustered with other plant-associated strains, such as *B. subtilis* 168. Additionally, strain Y31 exhibited ANIb values of 98.4% with *B. subtilis* DSM10 and *B. subtilis* 168, surpassing the standard 95% species delineation threshold (Jain et al., 2018), thereby confirming that strain Y31 is a member of the *B. subtilis* species. *B. subtilis* is widely recognized as a plant growth-promoting microorganism due to its ability to form highly resistant spores, its suitability for large-scale production, strong rhizosphere colonization capacity, and multiple plant growth-promoting mechanisms (Miljaković et al., 2020; Blake et al., 2021). Therefore, *B. subtilis* Y31, isolated from the cucumber rhizosphere, was applied to cucumber plants to evaluate its plant growth-promoting effects.

### 4.3 Potential impacts of *B. subtilis* Y31 on cucumber growth and soil properties

Previous reports using *B. subtilis* strains such as YB-04 (Xu et al., 2022), MBI600 (Samaras et al., 2020), and B579 (Fan et al., 2025) have demonstrated enhanced cucumber growth. However, relatively few studies have examined the effects of *B. subtilis* on cucumber yield. In this study, inoculation with *B. subtilis* Y31 significantly enhanced the growth of cucumber seedlings, including plant height, stem diameter, shoot fresh weight, and shoot dry weight compared to the CK group. Furthermore, *B. subtilis* Y31 significantly improved cucumber yield under greenhouse conditions, with average yield per plant increasing by 40.02% and total yield per plant by 35.30% relative to the CK group. These results suggested that *B. subtilis* Y31 was a promising plant growth-promoting bacterium.

Application of *B. subtilis* Y31 improved soil physicochemical properties. Inoculation with *B. subtilis* Y31 significantly increased soil TP and AP concentrations compared with the CK treatment (Table 1), which is consistent with its phosphorus-solubilizing ability during the cultivation. PSB function as biofertilizers by converting insoluble phosphorus into plant-available forms, thereby promoting phosphorus uptake and improving the efficiency of biological nitrogen fixation (Janati et al., 2021). Our results further showed that Y31 application significantly elevated soil TN and AN levels. Genomic analysis of strain Y31 revealed the presence of nitrite transport and reduction gene clusters (*nasABCDEF*), as well as the potassium uptake system (*ktrABCD*). However, no significant changes were observed in soil TK or AK levels following inoculation. The soil OM content also increased after Y31 application (Table 1), likely due to enhanced organic carbon degradation, supported by the presence of genes encoding carbohydrate-active enzymes within the Y31 genome (Figure 9). This was confirmed by the detection of amylase, cellulase, and β-mannanase activities during *B. subtilis* Y31 growth (Figure 2). Collectively, these findings indicate that *B. subtilis* Y31 effectively enhanced soil fertility.

### 4.4 Potential impacts of *B. subtilis* Y31 on soil microbial community

The application of microbial inoculants promotes dynamic interactions among functional microorganisms, soil matrices, and plant root systems, thereby enhancing nutrient assimilation and modulating rhizosphere microbial community composition (Trivedi et al., 2020). Previous studies have shown that inoculation with commercial *B. subtilis* products did not significantly increase greenhouse cucumber yield but did enhance soil microbial diversity (Wu et al., 2022). Additionally, *B. subtilis* B579 reportedly inhibited the growth of the pathogenic fungus *F. oxysporum*, improved soil physicochemical properties, and increased both bacterial and fungal community diversity (Fan et al., 2025). Similarly, application of *B. subtilis* K424 under higher nutrient solution concentrations significantly increased the diversity and abundance of bacterial communities in cucumber cultivation systems (Li et al., 2023).

In this study, Y31 inoculation exerted a more pronounced influence on fungal communities than on bacterial communities in the cucumber rhizosphere. Distinct dominant taxa were observed among treatments at both the phylum and genus levels (Figure 7). Notably, Y31 significantly promoted the increase in relative abundance of phyla and genera associated with cucumber growth, including the phylum *Basidiomycota* and the genus *Penicillium*. The relative abundance of the phylum *Basidiomycota* was significantly higher in Y31-treated samples compared to the control (Table 3). Previous studies indicate that members of this phylum participate in lignocellulose degradation and contribute to pathogen suppression (Schmidt-Dannert, 2016; Clericuzio et al., 2021). The relative abundance of *Basidiomycota* was also increased in cucumber rhizosphere under straw return and wheat green manure amendments, suggesting *Basidiomycota* played an important role as a beneficial microbial group in cucumber rhizosphere soil (Wang and Zhou, 2023; Yin et al., 2025). At the genus level, *Penicillium* was significantly enriched following Y31 application (Table 3). *Penicillium* species are known to secrete carbohydrate-degrading enzymes, such as cellulases and chitinases, which promote the breakdown of complex polysaccharides (Ning et al., 2024). In addition, they produce secondary metabolites with antimicrobial activity, effectively inhibiting pathogenic organisms (Nicoletti et al., 2007). Inoculation with *Penicillium* strains enhanced soil fertility, promoted cucumber growth, and provided protection against pathogens (Babu et al., 2015; Hossain et al., 2014). Conversely, Y31 inoculation significantly reduced the relative abundance of saprophytic fungi such as *Botryotrichum* and *Chrysosporium* (Table 3). Collectively, these shifts in rhizosphere fungal community composition enhance nutrient cycling and suppress soilborne pathogens, thereby improving cucumber growth performance.

### 4.5 Genomic analysis reveals the plant growth-promoting mechanism of *B. subtilis* Y31

Genomic analysis of strain Y31 identified 167 carbohydrate-active enzymes, including GHs, GTs, CEs, CBMs, PLs, and AAs (Figure 9), which are closely associated with plant growth promotion. These enzymes participate in the metabolism of carbohydrates and glycoconjugates, including lysozymes, chitinases, and glucosidases, facilitating the degradation of soil organic matter and the release of essential nutrients such as carbon, nitrogen, and phosphorus (Zerillo et al., 2013). Previous reports showed that the genomes of cucumber growth-promoting *Bacillus* strains also harbored a large number of carbohydrate-active enzymes (Xu et al., 2022; Yang et al., 2023), indicating that strain Y31 may possessed a robust capacity for rhizosphere carbon source utilization and transformation, which played a crucial role in its ability to promote cucumber growth. Additionally, numerous cucumber growth-promoting *Bacillus* strains possess the capacity to produce siderophores, including *B. velezensis* VJH504 (Yang et al., 2023), *B. thuringiensis* CR71 (Flores et al., 2020) and *B. subtilis* JNF2 (Yang S. Y. et al., 2024; Yang F. et al., 2024). In this study, we found that *B. subtilis* Y31 could synthesize siderophores that enhance plant iron acquisition while mitigating iron-induced toxicity, thereby contributing to improved plant health. The siderophore transport genes *yfiY, yfiZ, yfhA*, and *yusV* were identified within the Y31 genome (Supplementary Table 1). Moreover, gene clusters associated with chemotaxis, flagellar assembly, and motility—including seven *che* genes, three *flh* genes, three *flg* genes, fourteen *fli* genes, and two *mot* genes—were also detected. Similar clusters have been reported in other *Bacillus* strains and are strongly linked to root colonization capacity (Magno-Pérez-Bryan et al., 2015; Samaras et al., 2020). Thus, further investigation is warranted to clarify the root colonization mechanisms and interactions of strain Y31 in the rhizosphere.

In addition, strain Y31 exhibits strong potential as a biocontrol agent. Cucumber gray mold, caused by *B. cinerea*, is a major constraint in cucumber production (Samaras et al., 2021). Our results demonstrated that Y31 exerted antagonistic activity against *B. cinerea*, a fungal pathogen within the order *Helotiales* (Figure 2F). Correspondingly, taxonomic profiling revealed a reduced relative abundance of *Helotiales* in soil fungal communities following Y31 treatment. Y31 also synthesizes several hydrolytic enzymes, including proteases, cellulases, and β-mannanases, in pure culture (Figure 2), along with a diverse repertoire of carbohydrate-active enzymes that degrade fungal cell wall polymers and facilitate nutrient acquisition (Figure 9). Furthermore, inoculation with Y31 increased the relative abundance of beneficial microbial taxa while decreasing that of pathogenic groups (Table 3). Genomic analysis identified biosynthetic gene clusters associated with the production of antimicrobial secondary metabolites (Kaspar et al., 2019), including surfactin, bacillaene, fengycin, subtilosin A, and bacilysin (Table 6). Collectively, these findings indicate that *B. subtilis* Y31 enhances cucumber growth and contributes to disease suppression. However, further research is required to evaluate its biocontrol efficacy against diverse pathogens across different crops to fully assess its suitability for sustainable agricultural applications. Comprehensive characterization of its growth-promoting and biocontrol traits will support the development of Y31 as an effective microbial inoculant.

## 5 Conclusion

In conclusion, *B. subtilis* Y31, a phosphate-solubilizing bacterium isolated from the cucumber rhizosphere, significantly improves cucumber growth and yield. Inoculation with Y31 not only increased soil available phosphorus but also reshaped the composition and abundance of the soil microbial community. Genomic analysis confirmed that Y31 harbors functional gene clusters involved in phosphate solubilization and plant growth promotion. Therefore, *B. subtilis* Y31 represents a promising beneficial strain with strong potential for application in sustainable crop production systems.

## Data Availability

The original contributions presented in the study are publicly available. This data can be found here: BioProject accession PRJNA1288737, BioSample accession SAMN49861144 and GenBank accession PX761359.
