# Supplementary material for: Phosphate-solubilizing Bacillus subtilis Y31 promotes cucumber growth and yield: insights from rhizosphere microbiomics and bacterial genomics
Source: Front Microbiol. 2026 Jan 13;16:1751005. doi: 10.3389/fmicb.2025.1751005 (PMC12835394; doi:10.3389/fmicb.2025.1751005)
Supplement: Supplementary file 1 [file Data_Sheet_1.DOCX]

Supplementary Material


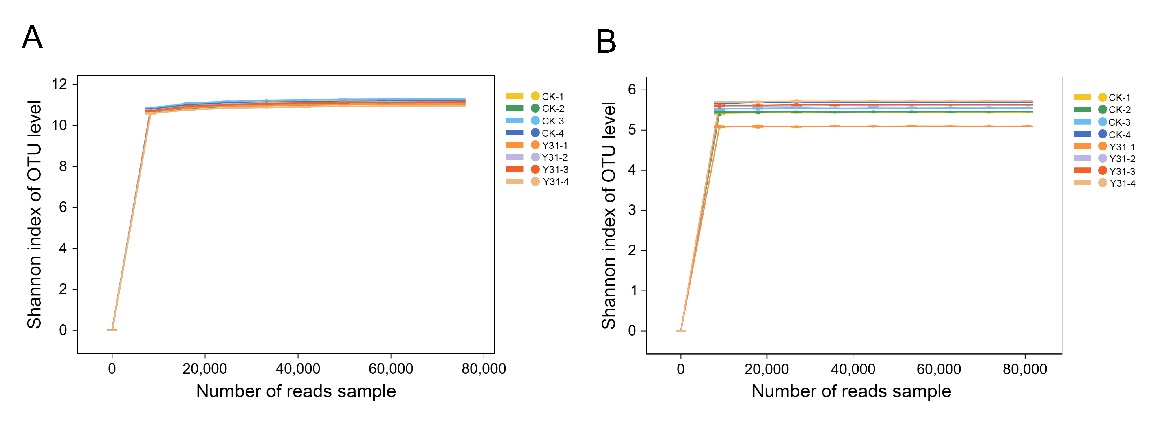


**Supplementary Figure 1.** The dilution curves of bacterial and fungal sequencing. **(A)** The dilution curves of bacteria; **(B)** the dilution curves of fungi.

**Supplementary Table 1.** Genes associated with plant growth promotion identified in the *B. subtilis* Y31 genome.

| **Function** | **Gene symbol** | **Location** | **SWISS ID** | **SWISS Description** |
| --- | --- | --- | --- | --- |
| Potassium assimilation | *ktrA* | 3,050,025-3,050,693 | O32080 | Ktr system potassium uptake protein A |
|  | *ktrB* | 3,050,700-3,052,037 | O32081 | Ktr system potassium uptake protein B |
|  | *ktrC* | 1,495,438-1,496,103 | P39760 | Ktr system potassium uptake protein C |
|  | *ktrD* | 1,392,776-1,394,125 | O31658 | Ktr system potassium uptake protein D |
|  | *nasF* | 349,603-351,048 | P42437 | Uroporphyrinogen-III C-methyltransferase |
|  | *nasE* | 351,115-351,435 | P42436 | Assimilatory nitrite reductase small subunit |
| Nitrate/nitrite assimilation | *nasC* | 351,467-353,884 | P42434 | Assimilatory nitrate reductase catalytic subunit |
|  | *nasD* | 354,005-356,137 | P42435 | Nitrite reductase |
|  | *nasB* | 356,144-358,459 | P42433 | Assimilatory nitrate reductase electron transfer subunit |
|  | *nasA* | 358,639-359,844 | P42432 | Nitrate transporter |
| Chemotaxis | *cheA* | 1,688,413-1,690,428 | P29072 | Chemotaxis protein CheA |
|  | *cheB* | 1,687,334-1,688,407 | Q05522 | Protein-glutamate methylesterase/protein-glutamine glutaminase |
|  | *cheW* | 1,690,449-1,690,919 | P39802 | Chemotaxis protein CheW |
|  | *cheC* | 1,690,938-1,691,567 | P40403 | CheY-P phosphatase CheC |
|  | *cheD* | 1,691,564-1,692,064 | P40404 | Chemoreceptor glutamine deamidase |
|  | *cheY* | 1,679,432-1,679,794 | P24072 | Chemotaxis protein CheY |
|  | *cheR* | 2,252,368-2,253,138 | P31105 | Chemotaxis protein methyltransferase |
| Siderophore | *yfiY* | 881,751- 882,728 | O31567 | Probable siderophore-binding lipoprotein YfiY |
|  | *yfiZ* | 882,859- 883,860 | O31568 | Probable siderophore transport system permease protein YfiZ |
|  | *yfhA* | 883,857- 884,888 | O31569 | Probable siderophore transport system permease protein YfhA |
|  | *yusV* | 3,240,714- 3,241,535 | O32188 | Probable siderophore transport system ATP-binding protein YusV |
| Flagellar assembly | *flgB* | 1,666,876-1,667,265 | P24500 | Flagellar basal body rod protein FlgB |
|  | *flgC* | 1,667,265-1,667,717 | P24501 | Flagellar basal-body rod protein FlgC |
|  | *fliE* | 1,667,722-1,668,048 | P24502 | Flagellar hook-basal body complex protein FliE |
|  | *fliF* | 1,668,109-1,669,704 | P23447 | Flagellar M-ring protein |
|  | *fliG* | 1,669,717-1,670,733 | P23448 | Flagellar motor switch protein FliG |
|  | *fliH* | 1,670,822-1,671,478 | P23449 | Probable flagellar assembly protein FliH |
|  | *fliI* | 1,671,475-1,672,791 | P23445 | Flagellum-specific ATP synthas |
|  | *fliJ* | 1,672,794-1,673,237 | P20487 | Flagellar FliJ protein |
|  | *fliK* | 1,673,876-1,675,339 | P23451 | Probable flagellar hook-length control protein |
|  | *flgG* | 1,675,780-1,676,574 | P23446 | Flagellar basal-body rod protein FlgG |
|  | *fliL* | 1,676,826-1,677,248 | P23452 | Flagellar protein FliL |
|  | *fliM* | 1,677,282-1,678,280 | P23453 | Flagellar motor switch protein FliM |
|  | *fliY* | 1,678,375-1,679,406 | P24073 | Flagellar motor switch phosphatase FliY |
|  | *fliZ* | 1,679,809-1,680,468 | P35536 | Flagellar biosynthetic protein FliZ |
|  | *fliP* | 1,680,575-1,681,126 | P35528 | Flagellar biosynthetic protein FliP |
|  | *fliQ* | 1,681,141-1,681,410 | P35535 | Flagellar biosynthetic protein FliQ |
|  | *fliR* | 1,681,418-1,682,197 | P35537 | Flagellar biosynthetic protein FliR |
|  | *flhA* | 1,683,312-1,685,345 | P35620 | Flagellar biosynthesis protein FlhA |
|  | *flhB* | 1,682,197-1,683,279 | P35538 | Flagellar biosynthetic protein FlhB |
|  | *flhF* | 1,685,345-1,686,445 | Q01960 | Flagellar biosynthesis protein FlhF |
|  | *motA* | 1,411,142-1,411,954 | P28611 | Motility protein A |
|  | *motB* | 1,410,385-1,411,068 | P28612 | Motility protein B |
